# Validation of maternal reports for low birthweight and preterm birth indicators in rural Nepal

**DOI:** 10.7189/jogh.08.010604

**Published:** 2018-06

**Authors:** Karen T Chang, Luke C Mullany, Subarna K Khatry, Steven C LeClerq, Melinda K Munos, Joanne Katz

**Affiliations:** 1 Department of International Health, Johns Hopkins University Bloomberg School of Public Health, Baltimore, Maryland, USA; 2 Nepal Nutrition Intervention Project–Sarlahi, Lalitpur, Nepal

## Abstract

**Background:**

Tracking progress towards global newborn health targets depends largely on maternal reported data collected through large, nationally representative surveys. We evaluated the validity, across a range of recall period lengths (1 to 24 months post-delivery), of maternal report of birthweight, birth size and length of pregnancy.

**Methods:**

We compared maternal reports to reference standards of birthweights measured within 72 hours of delivery and gestational age generated from reported first day of the last menstrual period (LMP) prospectively collected as part of a population-based study (n = 1502). We calculated sensitivity, specificity, area the under the receiver operating curve (AUC) as a measure of individual-level accuracy, and the inflation factor (IF) to quantify population-level bias for each indicator. We assessed if length of recall period modified accuracy by stratifying measurements across time bins and using a modified Poisson regression with robust error variance to estimate the relative risk (RR) of correctly classifying newborns as low birthweight (LBW) or preterm, adjusting for child sex, place of delivery, maternal age, maternal education, parity, and ethnicity.

**Results:**

The LBW indicator using maternally reported birthweight in grams had low individual-level accuracy (AUC = 0.69) and high population-level bias (inflation factor IF = 0.62). LBW using maternally reported birth size and the preterm birth indicator had lower individual-level accuracy (AUC = 0.58 and 0.56, respectively) and higher population-level bias (IF = 0.28 and 0.35, respectively) up to 24 months following birth. Length of recall time did not affect accuracy of LBW indicators. For the preterm birth indicator, accuracy did not change with length of recall up to 20 months after birth and improved slightly beyond 20 months.

**Conclusions:**

The use of maternal reports may underestimate and bias indicators for LBW and preterm birth. In settings with high prevalence of LBW and preterm births, these indicators generated from maternal reports may be more vulnerable to misclassification. In populations where an important proportion of births occur at home or where weight is not routinely measured, mothers perhaps place less importance on remembering size at birth. Further work is needed to explore whether these conclusions on the validity of maternal reports hold in similar rural and low-income settings.

Approximately 20 million low birth weight (LBW, <2500 grams) infants are born annually, the vast majority from low and middle income countries [1]. In South Asia 28% of infants are LBW, or one in every four births [2]. LBW is associated with increased mortality and morbidity, cognitive impairment, and long-term health complications in adulthood [1,3]. In 2012, one of six global nutrition targets set by World Health Organization Member States was to reduce LBW incidence by 30% by 2025 [4]. Each year, an estimated 15 million infants are born preterm, complications of which now constitute the leading cause of neonatal and under-five mortality [5-9]. More than 60% of preterm births occur in South Asia and Africa [7,8]. Preterm birth is associated with increased risk of cerebral palsy, vision and hearing impairment, and diminished learning abilities [7,8,10,11]. Preterm and LBW are linked but not synonymous conditions. Measuring and monitoring both LBW and preterm birth indicators is essential to tracking progress towards global targets to improve newborn health outcomes.

One challenge to accurately tracking LBW is that more than half of children globally, and up to 69% of children in South Asia, are not weighed at birth. Despite substantial progress, many births still occur at home [1,2,4] and are thus not measured. In facilities weight is inconsistently measured, and records are often incomplete and/or unreliable, and women delivering in facilities may differ from the broader population in important ways (for example, by health status, demographically, socio-economically) [1,2]. Measuring gestational age is also problematic in low-resource settings; routine ultrasound during the first trimester is not widely available, accessible or affordable [7]. This leads to reliance on reported date of last menstrual period (LMP), an error-prone measure given that collection is often late in pregnancy or at delivery, menstrual cycles vary in length, and non-negligible rates of lactational- or nutritional-amenorrhea exist in low resource settings [7].

Thus, maternally-reported information collected through national surveys, like the Demographic and Health Surveys (DHS) and the Multiple Indicator Cluster Surveys (MICS), is often utilized to generate birth indicators in low-income countries [12]. Recall periods are often long (i.e. up to five years prior to survey administration) [12]. Improved understanding of the validity of maternal reports of newborn health is critical for monitoring global newborn health targets. We assessed the validity of postpartum reports of birthweight, birth size and shortened length of pregnancy, by comparing maternal reports directly with data on birthweight and gestational age collected as part of a large community-randomized trial. By varying recall periods from 1 to 24 months, we examined if length of recall period modified indicator validity. We also assessed whether other maternal or newborn factors were associated with correct reporting of birthweight and preterm status.

## METHODS

### Study setting

The study was conducted in Sarlahi district of Nepal, bordering Bihar, India to the south. Among approximately 800 000 predominantly Hindu residents, 40% are less than 15 years of age [13]. Government census data indicate that 15% of married women wed prior to age 15 and approximately 55.8% and 36.6% of males and females, respectively, five years and older can read and write [13].

### Parent trial

Between November 2010 and January 2017, a randomized community-based trial enrolled pregnant women and their babies in 34 Village Development Committees in the rural district of Sarlahi, Nepal to investigate the impact of full-body newborn massage with sunflower seed oil on newborn deaths and infections. The trial was registered at ClinicalTrials.gov (NCT01177111). Locally-resident female study staff visited married women 15-35 years of age at home every 5 weeks and asked about date of last menstrual period in the past 5 weeks. If they had not menstruated in the past 5 weeks, they were offered a pregnancy test and if found to be pregnant, were asked if they would consent to enroll in the study. Enrolled women were followed through delivery; study staff visited as soon as possible after delivery and through the first month (days 1, 3, 7, 10, 14, 21, and 28). At the visit following birth, workers recorded date/time of delivery, circumstances of labor and delivery, health status of mother and newborn, and the median of three measures of baby’s weight using a digital scale precise to 10g (Tanita BD-585). The date, time of birth and weight of the newborn were also provided to the mother/caretaker on a small 10 × 8 centimeter card. Subsequent visits focused on maternal report and directly observed aspects of newborn health.

### Substudy

Between April and September 2016, mother/child pairs that had participated in the parent trial were selected for one additional follow-up visit to ask mothers to report on events during labor and delivery, immediate newborn care, postnatal care, and cases of illness and care sought in the first 7 days of life. Mothers who had a singleton live birth and whose visit following birth had been conducted within 72 hours after delivery were eligible. Given that a relatively low (i.e. <10%) proportion of newborns would have experienced illness or received postnatal care in the first week of life, we oversampled mother/child pairs with these characteristics. We defined illness as death or having two or more of the following signs in the same visit in the first week of life: difficulty sucking, difficulty breathing, stiffening of the back or convulsions, rapid breathing (a respiratory rate of 60 breaths per minute or faster), chest in-drawing, hyperthermia (100.4°F or higher), hypothermia (lower than 95.9°F), lethargy, or pus or redness at the base of the cord stump. We categorized newborns into four groups: those who experienced an illness, did not experience an illness, had a postnatal visit with a health provider outside of the study, and did not have a postnatal visit. All newborns enrolled in the study were visited (i.e. as part of the parent study procedures) in the immediate postnatal period by study staff, but not all had postnatal care visits with non-study providers. We sampled all newborns who experienced an illness and/or had a non-study postnatal care visit, and randomly sampled additional newborns without an illness and/or without a non-study postnatal visit. Rather than aim to produce a representative sample of the larger community, our intent was to ensure we could evaluate the accuracy of maternal recall for rarer events such as care seeking for newborn illness. We aimed to interview approximately equal numbers of different mothers at each of seven follow-up time periods: 1, 3, 6, 9, 12, 18, or 24 months after birth.

Study staff visited selected mothers and requested participation through an oral consent process in Nepali or Maithili, followed by collection of signature or thumbprint. Those agreeing were asked questions specific to this analysis (Table S1 in **Online Supplementary Document(Online Supplementary Document)**) in addition to questions about newborn care practices, morbidity and care seeking (results for those indicators will be published separately).

### Ethical approval

The study was approved by the Johns Hopkins Bloomberg School of Public Health Institutional Review Board in Baltimore, USA (parent trial and this substudy). In Nepal, approval was received from the Tribhuvan University Institute of Medicine, Kathmandu (parent trial) and the Nepal Health Research Council, Kathmandu (substudy).

### Data analysis

Here we focus on assessing the validity of maternal reports of A) birthweight and B) birth size in correctly categorizing newborns as LBW, and C) a shortened length of pregnancy in identifying preterm births. Maternal classification of LBW included a reported birthweight <2500 grams (regardless of gestational age) or birth size of “small” or “very small.” Maternal classification of preterm birth was defined as a reported time of delivery of “early” or “very early.” We compared the percent of babies classified as LBW using these maternal reports of birthweight and birth size, to the proportion so classified using weight data collected using the digital scale (i.e. within 72 hours of birth). Similarly, we compared maternal classification of preterm birth with our “gold standard” estimate of pregnancy length estimated by calculating the difference in days between delivery date and reported first day of the LMP, collected through prospective surveillance as described above.

Sensitivity, specificity, area under the curve (AUC), and the inflation factor (IF) were calculated. AUC is the area under the receiver operating characteristic curve (plot of 1 – specificity vs. sensitivity). An AUC of 1 represents a perfect test while a score of 0.5 suggests the test is no better than a random guess. The IF is the ratio of the survey-based prevalence of the indicator (given the estimated sensitivity and specificity) and the ‘true’ prevalence based on a gold standard, and thus quantifies the extent to which the indicator is over- or underestimated in a survey. The observed prevalence of an indicator in a population-based survey is equal to the true prevalence × (Sensitivity + Specificity – 1) + (1 – Specificity) [14]. Bootstrapping with 1000 replications was used to estimate standard errors and construct 95% confidence interval CIs for the IF ratio.

We stratified sensitivity, specificity, AUC, and IF by child sex, birth location (facility vs. home), maternal education (any vs. none), maternal age (<20 vs. ≥20 years) and parity (primiparous vs. multiparous). To examine whether accuracy of maternal reports erodes with longer recall periods, sensitivity, specificity, AUC, and IF were calculated within each of the seven bins of recall time. Comparable to other studies that have assessed validity of indicators, we defined *a priori* high individual-level accuracy as AUC>0.70, and low population-level bias as 0.75<IF<1.25 [15-17].

Finally, to control for possible differences in the types of mothers interviewed across recall periods, we used a modified Poisson regression with robust error variance to estimate relative risk ratios (RR) [18] to assess time since birth as the predictor on the outcome of mothers correctly identifying their newborns as LBW versus not LBW or preterm versus not preterm, controlling for child age, child sex, place of delivery, maternal age, maternal education, parity, and ethnicity. Stata version 14.0 (StataCorp, College Station, TX, USA) was used for the analyses.

## RESULTS

**Figure 1:**
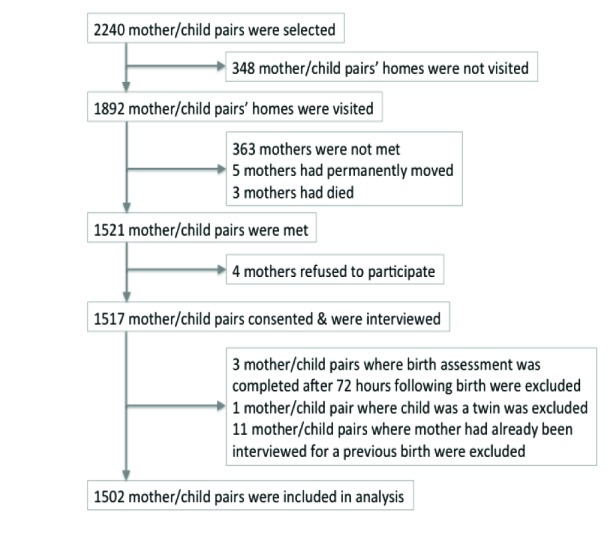
Flowchart of participant selection. Flowchart describing the process of the selection of participants included in this analysis.

Of the 1892 households visited, 363 (19%) mothers were not met at their homes. We consented and interviewed 1517 mothers (**Figure 1**). No differences were observed in the mothers interviewed compared to those not met by child sex, place of delivery, maternal age and maternal education, likely limiting the presence of selection bias. After excluding 15 participants (birth assessment >72 hours after birth [n = 3], twin delivery [n = 1], repeat participation [n = 11]), a total of 1502 mother/child pairs were included. Of these, 220 were enrolled in the one month recall group, 207 in the three month group, 205 in the six month group, 194 in the nine month group, 193 in the 12 month group, 284 in the 18 month group, and 199 in the 24 month group. The mean recall period was 10.7 months (Table S2 in **Online Supplementary Document**). More than half of newborns were male (55.5%) and a majority of these births occurred in the home (53.7%). The mean age of mothers at follow up was 23.8 years; most had no schooling (68.2%) and had prior children (71.4%). Participants were nearly universally (96.2%) of Madhesi ethnic origin, frequently lacked a household latrine (71.2%), had electricity (80.5%), and owned some type of land (97.4%). Our sample was largely comparable to the parent trial sample; one difference was that the parent trial sample was more balanced by child sex (male = 51.3%). The median gestational age at the time pregnancy was first identified was comparable between the parent trial and this substudy: 14.3 weeks (interquartile range IQR = 9.7-23.9 weeks) and 14.6 weeks (IQR = 9.9-23.1 weeks), respectively.

**Figure 2:**
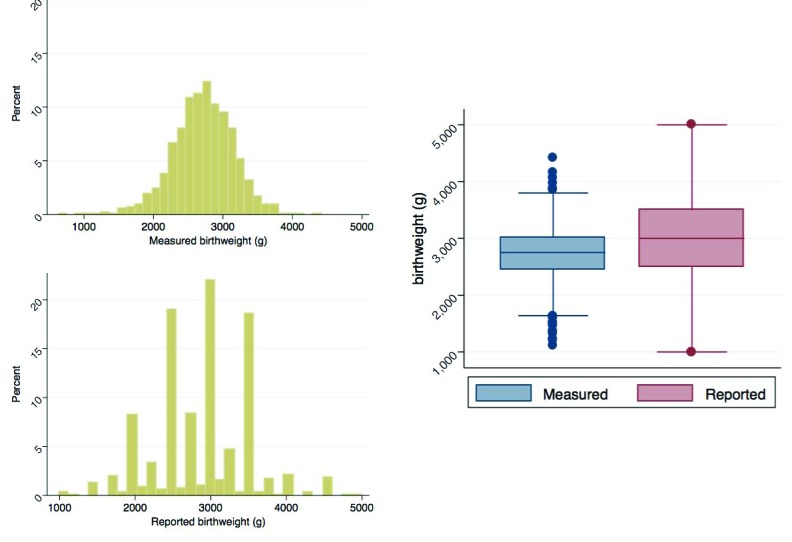
Distributions of measured and reported birthweight. Histograms and boxplots of the distribution of measured and reported birthweights.

**Figure 3:**
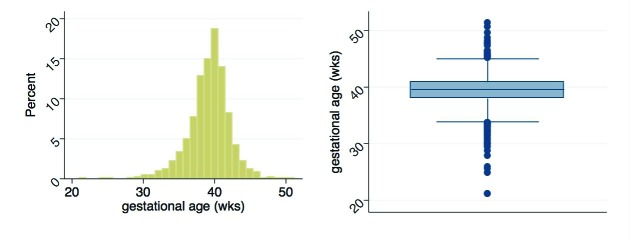
Distribution of gestational age. Histogram and boxplot of the distribution of gestational age calculated from the first day of the last menstrual period and the date of delivery collected in the parent trial.

Mother/child pairs missing either the actual digital weight measures or the maternal assessment of weight were excluded. The former included deaths prior to measurement (n = 14), parental refusal of weight measurement (n = 1), and missing weight measurement (n = 1); the latter exclusions included pairs where mothers reported the child was not weighed (n = 21), was uncertain if child was weighed (n = 6), or was weighed but could not respond (n = 47). A further six mothers without LMP data were excluded. **Figure 2** provides the distributions of measured and reported birthweights, and Figure 3 describes the distribution of calculated gestational age. Measured birthweights and calculated gestational age appear to be normally distributed with medians at 2750g (IQR = 2460-3000g) and 39.6 weeks (IQR = 38-40.9 weeks), respectively. Reported birthweights are heavily heaped, and have a higher median (3000g) and a larger spread (IQR = 2500-3500g) compared to measured birthweights. Results in **Table 1** are analogous with a greater mean and standard deviation (SD) in reported birthweights (mean = 2886g, SD = 608g) compared to measured birthweights (mean = 2726g, SD = 435g). A higher percentage of newborns were categorized as LBW when using the measured values (27.6%, 95% CI = 25.4-30.0%) compared to the reported values (17.1%, 95% CI = 15.3-19.2%). This pattern is generally consistent when examining birthweight and percent LBW by sex and by birth size. Within both measured and reported values, mean birthweight increased and the percentage of newborns identified as LBW decreased with increasing birth size, though the trend was not statistically significant. Mean calculated gestational age was 39.3 weeks (SD = 2.9 weeks) with 16.1% (95% CI = 14.3-17.9%) of newborns categorized as preterm, comparable by sex. Though not statistically significant, mean gestational age increased with increased reported length of pregnancy and the percentage of preterm births decreased with the exception of the late and very late groups.

**Table 1:** Summary of birthweight, gestational age, low birth weight, and preterm birth

	Measured birthweight			Reported birthweight				Gestational age (weeks)		
	**N**	**Mean (SD) grams**	**LBW (< 2500g) % (95% CI)**	**N**	**Mean (SD) grams**	**LBW (< 2500g) % (95%CI)**		**N**	**Mean (SD)**	**Preterm (<37 weeks) % (95% CI)**
**All**	1486	2726 (435)	27.6 (25.4, 30.0)	1429	2886 (608)	17.1 (15.3, 19.2)	**All**	1496	39.3 (2.9)	16.1 (14.3, 17.9)
**Sex:**							**Sex:**			
Male	826	2792 (445)	23.1 (20.4, 26.1)	790	2928 (610)	16.5 (14.0, 19.2)	Male	829	39.2 (3.0)	16.6 (14.3, 19.3)
Female	660	2644 (409)	33.2 (29.7, 36.9)	639	2834 (602)	18.0 (15.2, 21.2)	Female	667	39.4 (2.8)	15.3 (12.8, 18.2)
**Birth size:**							**Birth timing:**			
Very small	30	1945 (622)	80.0 (61.7, 90.9)	26	2060 (818)	69.2 (49.0, 84.0)	Very early	22	35.2 (5.3)	59.1 (37.7, 77.5)
Small	83	2297 (413)	63.8 (53.0, 73.5)	77	2221 (470)	66.2 (54.9, 75.9)	Early	61	37.4 (3.1)	36.1 (25.0, 48.9)
Average	1252	2748 (387)	25.1 (22.8, 27.6)	1211	2904 (554)	13.7 (11.9, 15.8)	On time	1270	39.3 (2.8)	15.5 (13.6, 17.6)
Large	103	2990 (480)	16.5 (10.5, 25.0)	101	3306 (617)	8.9 (4.7, 16.3)	Late	115	40.9 (2.1)	3.5 (1.3, 8.9)
Very large	16	3078 (334)	6.3 (0.8, 35.1)	13	3577 (793)	0	Very late	22	41.0 (3.6)	9.1 (2.2, 30.7)
Don't know	2	2430 (156)	50.0 (1.9, 98.1)	1	2000 (-)	1	Don't know	6	37.2 (0.9)	33.3 (7.2, 76.3)

SD – standard deviation, LBW – low birthweight, CI – confidence interval

Of the 1476 mothers who were asked if they had a card with a birthweight record, only 74 (9.4%) of those who delivered at home and 53 (7.7%) of those who delivered at a facility were able to produce one (Table S3 in **Online Supplementary Document(Online Supplementary Document)**). Of the total of 127 cards presented, 22 were from a facility and 105 were from the parent study. Of the 22 facility cards, all had reported delivering at a facility, and of the 105 birth cards distributed during the parent trial, 70.5% delivered in the home and 29.5% delivered in a facility. Comparing the percent of newborns that would be categorized as low birthweight based on birthweight measurements taken during the parent trial versus the birth cards produced at the follow up visit, fewer would be categorized as low birthweight for home births (17.6% vs. 20.3%) and more would be identified as low birthweight among facility births (26.4% vs. 22.6%). This observation is purely descriptive as no statistical test was performed with so few birth cards presented.

Sensitivity, specificity, AUC, and IF are presented in **Table 2** for the following indicators: A) LBW newborns based on maternally reported birthweight, B) LBW newborns based on maternally reported birth size, and C) preterm births based on maternally reported length of gestation (absolute numbers available in Table S4 in **Online Supplementary Document(Online Supplementary Document)**). Sensitivity for all three indicators was low while specificity was high. LBW, estimated from maternal reports of birthweight, had low individual reporting accuracy (AUC = 0.69, 95% CI = 0.67-0.72) and high population-level bias (IF = 0.62, 95% CI = 0.52-0.72). Using reports of birth size and pregnancy length to estimate LBW and preterm birth prevalence also resulted in low individual-level accuracy (AUC = 0.58, 0.56) and high population-level bias (IF = 0.28, 0.35), respectively.

**Table 2:** Overall sensitivity, specificity, AUC, IF

Indicator	n	Sensitivity (%) (95% CI)	Specificity (%) (95% CI)	AUC (95% CI)	“True” prevalence (%) (95% CI)	Estimated survey-based prevalence (%) (95% CI)	IF (95% CI)
LBW using reported birthweight	1424	45.0 (40.0-50.1)	93.5 (91.8-94.9)	0.69 (0.67-0.72)	27.3 (25.0-30.0)	17.0 (15.1-19.1)	0.62 (0.52-0.72)
LBW using reported birth size	1486	19.1 (15.4-23.2)	96.7 (95.4-97.7)	0.58 (0.56-0.60)	27.7 (25.5-30.1)	7.7 (6.4-9.1)	0.28 (0.22-0.34)

CI – confidence interval, AUC – area under the receiver operating curve, IF – inflation factor, LBW – low birthweight, PTB – pre-term birth

To further investigate population-level bias, we plotted the values of the predicted survey prevalence of each of the three indicators across all possible prevalences of LBW and preterm births within our reference standard (Figure S1 in **Online Supplementary Document(Online Supplementary Document)**) [17]. We would not expect most populations to have a prevalence of LBW and preterm newborns in the high ranges; therefore, this figure is for illustrative purposes. The gray dotted line represents perfect reporting accuracy with 100% sensitivity and specificity across all possible prevalences within our reference standard. The red, blue and green lines show the estimated survey-based prevalence and the differences in the predicted survey prevalence and the ‘true’ prevalence using the estimated levels of sensitivity and specificity for each indicator. All three indicators, with low sensitivity and high specificity, underestimate the survey-based prevalence in our study population, and would underestimate the survey-based prevalence to a greater degree in populations with higher prevalence of LBW and preterm births. In lower-prevalence populations, the bias would be lower. Assuming sensitivity and specificity remain the same as prevalence changes, survey-based estimates would underestimate the magnitude of changes in prevalence when looking at time trends or across countries. Stratified analyses by child sex, place of delivery, any maternal education, maternal age, and parity did not generally produce significantly different results (Table S5 in **Online Supplementary Document(Online Supplementary Document)**).

**Figure 4:**
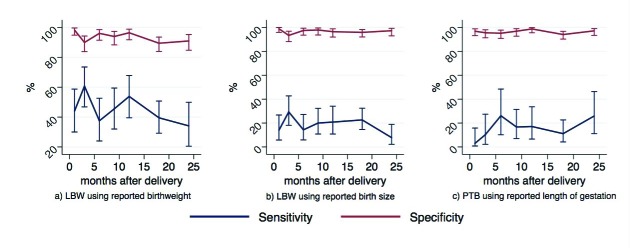
Sensitivity and specificity of LBW and preterm births over recall time. Sensitivity and specificity of **A**) LBW using reported birthweight, **B**) LBW using reported birth size, C) preterm birth using reported length of gestation over binned recall time.

We observed no significant differences in the measures of accuracy by binned recall time for any of the three indicators (**Figure 4** and Tables S6-8 in **Online Supplementary Document(Online Supplementary Document)**). These findings were consistent with our estimated RR for the proportions accurately categorized as LBW and preterm against recall length, controlling for child sex, place of delivery, maternal age, maternal education, parity, and ethnicity (Tables 3-4). In Model A, the estimated RR for the proportion of newborns correctly identified as LBW by maternally-reported birthweight negligibly decreased with increasing length of recall time and was statistically significant (RR = 0.99, 95% CI = 0.99-1.00, *P* = 0.004), adjusting for other factors. In Model B, the estimated RR for the proportion of newborns correctly identified as LBW by maternally-reported birth size similarly decreased only slightly with increasing length of recall time but was not statistically significant (RR = 0.99, 95% CI = 0.99-1.00, *P* = 0.10), controlling for other variables. For both models, mothers’ reports were less likely to accurately identify newborns as LBW if their child was female, and were more likely to accurately categorize the child if the mother reported having had any education and if the mother had one or more children prior to the child of interest. Model C included an inflection point at 20 months in the time since birth. Up to 20 months after birth, the estimated RR for the proportion of newborns correctly identified as preterm by maternally reported length of gestation was not associated with time (RR = 1.00, 95% CI = 0.99-1.00, *P* = 0.14), adjusting for other variables. After 20 months since birth, the estimated RR for the proportion of newborns correctly identified as preterm by maternally reported length of gestation improved slightly with increasing time since birth and was statistically significant (RR = 1.03, 95% CI = 1.01-1.05, *P* = 0.003). Maternal age, parity, and ethnicity were also predictive of correct categorization of preterm birth.

**Table 3:** Modified Poisson regression with robust error variance to estimate RR of correctly classifying newborns as A) LBW using maternally-reported birthweight, B) LBW using reported birth size

	A				B			
	**n**	**aRR**	**95% CI**	***P*-value**	**n**	**aRR**	**95% CI**	***P*-value**
**Time since birth/child age (months)**	1423	0.99	(0.99, 1.00)	0.004	1484	0.99	(0.99, 1.00)	0.10
**Child sex:**								
Male (ref)	788				825			
Female	635	0.93	(0.88, 0.98)	0.005	659	0.89	(0.84, 0.95)	<0.001
**Place of delivery:**								
Home (ref)	760				796			
Facility	663	0.95	(0.90, 1.01)	0.09	688	0.95	(0.90, 1.02)	0.16
**Maternal age (years)**	1423	1.00	(0.99, 1.01)	0.32	1484	1.00	(1.00, 1.01)	0.26
**Maternal education:**								
None (ref)	959				1011			
Any	464	1.06	(1.00, 1.11)	0.04	473	1.09	(1.02, 1.16)	0.01
**Parity:**								
Primiparous (ref)	411				422			
Second child	367	1.09	(1.01, 1.17)	0.04	378	1.20	(1.10, 1.32)	<0.001
Third child	291	1.09	(1.00, 1.19)	0.05	310	1.23	(1.11, 1.36)	<0.001
Fourth child or greater	354	1.07	(0.96, 1.19)	0.27	374	1.21	(1.07, 1.36)	0.002
**Ethnicity:**								
Pahadi (ref)	57				57			
Madhesi	1366	1.02	(0.89, 1.17)	0.77	1427	0.96	(0.83, 1.11)	0.58

aRR – adjusted relative risk, CI – confidence interval, LBW – low birthweight

**Table 4:** Modified Poisson regression with robust error variance to estimate RR of correctly classifying newborns as C) preterm birth using reported shortened length of gestation

	C			
	**n**	**aRR**	**95% CI**	***P*-value**
**Time since birth/child age (1-20 months)**	1494	1.00	(0.99, 1.00)	0.14
**Time since birth/child age (>20 months)**	1494	1.03	(1.01, 1.05)	0.003
**Child sex:**				
Male (ref)	828			
Female	666	1.01	(0.96, 1.05)	0.76
**Place of delivery:**				
Home (ref)	802			
Facility	692	1.04	(0.99, 1.09)	0.16
**Maternal age (years)**	1494	0.99	(0.98, 1.00)	0.003
**Maternal education:**				
None (ref)	1018			
Any	476	1.01	(0.96, 1.07)	0.63
**Parity:**				
Primiparous (ref)	429			
Second child	379	1.09	(1.02, 1.16)	0.01
Third child	310	1.14	(1.06, 1.23)	0.001
Fourth child or greater	376	1.11	(1.00, 1.22)	0.04
**Ethnicity:**				
Pahadi (ref)	57			
Madhesi	1437	0.88	(0.81, 0.96)	0.002

aRR – adjusted relative risk, CI – confidence interval

## DISCUSSION

Accurate LBW and preterm estimates are necessary for assessing prevalence and denominators of indicators to assess coverage of interventions aimed at improving neonatal outcomes. We found that maternal reporting of birthweight, birth size, and length of gestation underestimates the true prevalence of LBW and preterm birth. LBW using maternally reported birthweight had low individual-level accuracy and high population-level bias up to 24 months following birth. Several studies in high-income settings have demonstrated high accuracy of maternal recall of birthweight compared to hospital records [19-23]. However, one study in Taiwan where mothers were asked via phone to recall their child’s birthweight, mothers tended to overestimate birthweight and found maternal recall used to categorize children as LBW had low sensitivity (52%) and high specificity (95.3%) [24]. This is consistent with our findings though children in Taiwan were much older at the time of follow-up. In low-income settings, results have been heterogeneous. Findings from a validation study in Kenya of maternal recall of LBW at hospital discharge after birth and 13-15 months after birth compared to directly-observed deliveries found high reporting accuracy and low population-level bias at both time points [17,25]. A study in Colombia assessing maternal recall for LBW 5-12 years after birth reported high specificity (95%) but low sensitivity (66%) [26]. Similar to findings from Taiwan, a study in Uganda described mothers over-reporting birthweight 4-7 years after delivery [27] while another study in Brazil found that mothers of children who weighed less than 3500g at birth tended to overestimate birthweight while those with children weighing more tended to underestimate birthweight 11 years after delivery [28]. One possible reason for the lower sensitivity and higher degree of overestimating birthweight in our study compared to other studies may be partially explained by the relatively smaller size of newborns in this rural Nepal population. With more babies clustered around the cutoff of less than 2500g, we observed a high likelihood of misclassification in a population with high prevalence of LBW (27.7%).

We found the LBW indicator based on reported birth size had both low individual-level accuracy and high population-level bias. This indicator in our study had much lower sensitivity and higher specificity (Sp) compared to that described in a study in Uganda (Sn = 76%, 95%CI: 50-93% and Sp = 70%, 95%CI: 65-75%) [27]. Other studies that assessed the relationship of birthweight and birth size within DHS datasets have found that mean birthweight generally decreased with decreasing birth size, consistent with our findings [29-32]. However, when using maternal recall of birth size as an indicator for LBW, sensitivity was low while specificity was high [29-32]. All such studies acknowledged that these analyses were limited by selection bias in that mothers who were able to report a birthweight were more likely to have delivered in a facility and be of higher socioeconomic status. Our study results demonstrate similar findings to these prior studies in a population with more than half of deliveries occurring in the home. Channon describes mothers’ perception of birth size as being influenced by various neighborhood and regional factors within a societal context that frames a reference for how mothers gauge their child’s size [33]. Applied to our study population, children in this community relative to the global context are generally smaller perhaps leading mothers to gauge smaller children as being of average size.

We observed both low individual-level accuracy and high population-level bias for the preterm birth indicator generated from maternal reports of length of gestation at birth. Several studies have reported high degrees of accuracy of gestational age reports from mothers in developed countries [19,21,22,34,35]. One study conducted in the US Nurses’ Health Studies population reported moderate sensitivity (68%) and high specificity (92%) using maternally reported gestational age to classify preterm birth [20]. Our study is limited in that we did not ask mothers to provide a numerical estimate of gestational age and have only reported the validity of using categories of gestational length at birth. We modeled this question after the format of the birth size question used in the DHS and MICS surveys, and since this question has not been used outside of this study, we recommend it be further refined before use in other settings.

Contrary to our hypothesis, the length of time since delivery did not affect the validity of maternal report for LBW and longer length of time among mothers we visited 20 months since birth and later resulted in improved accuracy for preterm birth. Some studies have reported improved accuracy and agreement between medical records and maternal report for birthweight and gestational age associated with shorter length of recall [21,34,36] while others have found accuracy of maternal report does not significantly deteriorate over time [20,24,26]. All these studies investigated patterns over longer periods of time spanning years rather than months, which limits comparability to our study findings. We observed a slightly lower degree of accuracy of maternal report used to correctly classify LBW for female compared to male children, which was not observed in other studies in developing countries [26,27]. Researchers have documented the association of sex biases in neonatal care-seeking, household food allocation and higher mortality among girls compared to boys and the persistence of son preferences in this community [37] and elsewhere in South Asia [37-39]. Further work is needed to explore whether this bias may be applicable to the accuracy of maternal report in this setting. We also observed slight improvements in maternal report accuracy associated with maternal education, consistent other studies’ findings [19,22,26,27]. Finally, across all three indicators, we found that multiparous mothers had greater accuracy compared to first-time mothers, which contrasts with patterns described in prior studies [19,26,27,35].

A strength of our study is the inclusion of home births in a population with relatively high prevalence of LBW and preterm birth. In the South Asian region, where around 69% of newborns are not weighed at birth, perhaps mothers place less importance on remembering and documenting birthweight, as evidenced by very few mothers who were able to present a birthweight card. Also, our study used accurate and calibrated scales of research quality and trained and supervised data collectors, in contrast to many delivery facilities. We also demonstrated that these indicators may be increasingly vulnerable to being underestimated in populations with higher prevalences. A limitation of our birthweight measurements used as the gold standard is that newborns were weighed up to 72 hours after birth. In this time period, newborns normally lose weight before patterns of growth and weight gain are observed. Therefore, our measurements were likely taken at a nadir and overestimate the prevalence of LBW; however, our intention was to validate maternal report rather than provide an estimate of prevalence. In addition, for home births, we are fairly confident that mothers were reporting the birthweight measured during the parent trial since this would have been the only birthweight provided to them. However, mothers who delivered in a facility may have had their child weighed both at the facility and during participation in the parent trial. For facility births, we assumed the mother was reporting the weight measurement during the parent trial. Lastly, we did not ask mothers to report birthweight immediately after the measurement taken, which would have provided more information about whether mothers could retain birthweight information if the event occurred just prior to our interview.

Our conclusions regarding appropriate classification of preterm birth are limited since we did not ask mothers to report a numerical length of gestational age. Our categories of gestational length at birth were adapted from the DHS and MICS birth size question and have only been used in this study. Additionally, our gold standard for gestational age is based only on reported LMP, which frequently overestimates gestational age by a few days when compared to the gold standard of ultrasound measurements taken in the first trimester [40]. Our reliance on this error-prone measure may have led to misclassification of preterm births, an important limitation of our study. However, in low-income settings, ultrasound is generally not feasible. Our reported LMP was collected within a five-week period during pregnancy in order to optimize accurate recall, but we recognize this date is likely subject to errors in reporting.

## CONCLUSIONS

The use of maternal reporting may underestimate and bias indicators for LBW and preterm birth. Additional approaches may be needed to correct for these inaccuracies in large surveys and improve methods to monitor and track progress towards global newborn health targets. The findings of this study may have limited generalizability to settings with high prevalence of LBW and preterm births and where the majority of births take place in the home. Further work is needed to explore whether these conclusions on the validity of maternal reporting hold in similar rural and low-income settings. Additional studies may be needed to understand the range of likely individual-level accuracy and population bias for these indicators in settings where women more commonly deliver in facilities and health cards are more frequently retained.
