# Supplementary material for: Validation of maternal reports for low birthweight and preterm birth indicators in rural Nepal
Source: J Glob Health. 2018 Jun 9;8(1):010604. doi: 10.7189/jogh.08.010604 (PMC5997365; doi:10.7189/jogh.08.010604)
Supplement: Online Supplementary Document [file jogh-08-010604-s001.pdf]

Online Supplementary Document

Chang et al. Validation of maternal reports for low birthweight and preterm birth indicators in rural Nepal

J Glob Health 2018;8:010604

Title: Validation of maternal reports for low birthweight and preterm birth indicators in rural Nepal

Online Supplementary Document

List of Supplementary Tables and Figures:

- Table S1: Birthweight, birth size, and gestational age birth timing questions administered
- Table S2: Characteristics of selected mothers & children
- Table S3: Birthweight cards by place of delivery
- Table S4: 2x2 tables for low birthweight and preterm birth indicators
- Table S5: Sensitivity, specificity, AUC stratified by child sex, place of delivery, maternal education, maternal age, and parity
- Table S6: Sensitivity, specificity, AUC for LBW using reported birthweight by recall group
- Table S7: Sensitivity, specificity, AUC, IF for LBW using reported birth size by recall group
- Table S8: Sensitivity, specificity, AUC, IF for preterm using reported birth timing by recall group
- Figure S1: Inflation factors.

Table S1: Birthweight, birth size, and gestational age birth timing questions administered

	Question	Answer choices
1	When your child was born, was he/she born very early, early, on time, late, or very late?	1=Very early<br>2=Early<br>3=On time<br>4=Late<br>5=Very late<br>9=Don't know
2	When your child was born, was he/she very large, larger than average, average, smaller than average, or very small?	1=Very small<br>2=Smaller than average<br>3=Average<br>4=Larger than average<br>5=Very large<br>9=Don't know
3	Was your child weighed at birth?<br>(Within 1 hour after birth; including a Balposan worker)	0=No (Go to 3a)<br>1=Yes (Go to 3b)<br>9=Don't Know (Go to 3a)
3a.	Was your child weighed within 3 days after birth?	0=No (Go to next question)<br>1=Yes (Go to 3b)<br>9=Don't Know (Go to next question)
3b.	How much did your child weigh the first time they were weighed?<br><br>(ASK MOTHER TO RECALL ONLY; DO NOT USE BIRTH RECORD OR CERTIFICATE)	(gm)______________<br><br>9999=Don't Know
3c.	Do you have a birth record or certificate with your child's birth weight recorded the first time they were weighed?	0=No (Go to next question)<br>1=Yes (Go to 3d)<br>9=Don't Know (Go to next question)
3d.	(CHECK THE BIRTH RECORD OR CERTIFICATE AND RECORD THE CHILD'S BIRTHWEIGHT. RECORD WHETHER HOSPITAL OR PARENT TRIAL CARD)	(gm) ______________<br><br>1=Hospital card<br>2=Parent trial card

Table S2: Characteristics of selected mothers & children

	n	% or Mean	95% CI
**Child Age (months)**	1502	10.7	(10.3, 11.1)
**Child Sex**	1502		
Male	834	55.5	(52.9, 58.0)
Female	668	44.5	(42.0, 47.0)
**Place of delivery**	1501		
Home	806	53.7	(51.2, 56.2)
Facility	695	46.3	(43.8, 48.8)
**Maternal Age (yrs)**	1502		
<19	252	16.8	(14.9, 18.8)
20-29	1036	69.0	(66.6, 71.3)
30-39	197	13.1	(11.5, 14.9)
>40	17	1.1	(0.0, 1.8)
**Maternal Education**	1502		
No Schooling	1025	68.2	(65.8, 70.6)
Any Schooling	477	31.8	(29.4, 34.2)
**Parity**	1502		
primiparous	430	28.6	(26.4, 31.0)
second	381	25.4	(23.2, 27.6)
third	311	20.7	(18.7, 22.8)
fourth or higher	380	25.3	(23.2, 27.6)
**Ethnicity**	1501		
Pahadi	57	3.8	(2.9, 4.9)
Madhesi	1444	96.2	(95.1, 97.1)
**HH latrine status**	1501		
No latrine	1068	71.2	(68.8, 73.4)
Had latrine	433	28.8	(26.6, 31.2)
**HH electricity**	1501		
No electricity	293	19.5	(17.6, 21.6)
Had electricity	1208	80.5	(78.4, 82.4)
**Land ownership**	1501		
Did not own land	39	2.6	(1.8, 3.5)
Owns land	1462	97.4	(96.5, 98.1)

Table S3: Birthweight cards by place of delivery

	Total<br>N	Home<br>n (%)	Facility<br>n (%)
Mother was able to present a card with a birthweight	1476	74 (9.4)	53 (7.7)
Card was from:			
Facility	22	0	22 (100)
Study	105	74 (70.5)	31 (29.5)
Low birth weight using measured birthweight	127	13 (17.6)	14 (26.4)
Low birth weight using birthweight on card	127	15 (20.3)	12 (22.6)

Table S4: 2x2 tables for low birthweight and preterm birth indicators

A) Low birth weight using reported birthweight				
		LBW by measured birthweight		
		Yes	No	
LBW by reported birthweight	Yes	175	68	243
	No	213	968	1181
		388	1036	1424

B) Low birth weight using reported birth size				
		LBW by measured birthweight		
		Yes	No	
LBW by reported birth size	Yes	77	36	113
	No	333	1040	1373
		410	1076	1486

C) Preterm using reported birth timing				
		Preterm by calculated gestational age		
		Yes	No	
Preterm by reported length of pregnancy	Yes	35	48	83
	No	205	1208	1413
		240	1256	1496

Table S5: Sensitivity, specificity, AUC stratified by child sex, place of delivery, maternal education, maternal age, and parity

Indicator	Total N	Sensitivity	(95% CI)	Specificity	(95% CI)	AUC	(95% CI)
**Low birth weight using reported birthweight**	1424	45.0%	[40.0 - 50.1]	93.5%	[91.8 - 94.9]	0.69	[0.67 - 0.72]
By child sex							
Male	788	48.9%	[41.4 - 56.4]	93.1%	[90.9 - 95.0]	0.71	[0.67 - 0.75]
Female	636	41.7%	[35.0 - 48.7]	94.0%	[91.3 - 96.0]	0.68	[0.64 - 0.71]
By place of delivery							
Home	663	38.9%	[32.2 - 46.0]	94.8%	[92.4 - 96.7]	0.67	[0.63 - 0.70]
Facility	760	51.6%	[44.2 - 58.9]	92.4%	[89.9 - 94.4]	0.72	[0.68 - 0.76]
By maternal education							
None	959	47.6%	[41.6 - 53.7]	92.2%	[90.0 - 94.1]	0.70	[0.67 - 0.73]
Any	465	38.8%	[29.9 - 48.3]	96.0%	[93.4 - 97.8]	0.67	[0.63 - 0.72]
By maternal age							
<20 yrs	239	46.9%	[36.6 - 57.3]	94.4%	[89.3 - 97.6]	0.71	[0.65 - 0.76]
20+ yrs	1185	44.4%	[38.6 - 50.3]	93.3%	[91.5 - 94.9]	0.69	[0.66 - 0.72]
By parity							
Primiparous	412	46.4%	[38.6 - 54.3]	94.4%	[90.7 - 96.9]	0.70	[0.66 - 0.74]
2 or more children	1012	44.0%	[37.4 - 50.8]	93.2%	[91.2 - 94.9]	0.69	[0.65 - 0.72]
**Low birth weight using reported birth size**	1486	19.1%	[15.4 - 23.2]	96.7%	[95.4 - 97.7]	0.58	[0.56 - 0.60]
By sex							
Male	826	20.2%	[14.8 - 26.6]	96.6%	[94.8 - 97.8]	0.58	[0.56 - 0.76]
Female	660	18.1%	[13.3 - 23.8]	96.9%	[94.8 - 98.3]	0.58	[0.56 - 0.60]
By place of delivery							
Home	688	17.8%	[12.9 - 23.7]	96.4%	[94.4 - 97.9]	0.57	[0.54 - 0.60]
Facility	797	20.4%	[15.1 - 26.6]	96.9%	[95.1 - 98.1]	0.59	[0.56 - 0.62]
By maternal education							
None	1012	21.3%	[16.8 - 26.4]	96.4%	[94.8 - 97.6]	0.59	[0.56 - 0.61]

Any	474	13.6%	[8.0 - 21.1]	97.2%	[94.9 - 98.6]	0.55	[0.52 - 0.59]
By maternal age							
<20 yrs	249	20.8%	[13.4 - 30.0]	96.0%	[91.4 - 98.5]	0.58	[0.54 - 0.63]
20+ yrs	1237	18.5%	[14.4 - 23.3]	96.8%	[95.4 - 97.8]	0.58	[0.55 - 0.60]
By parity							
Primiparous	423	17.8%	[12.3 - 24.4]	96.5%	[93.4 - 98.4]	0.57	[0.54 - 0.60]
2 or more children	1063	20.0%	[15.2 - 25.6]	96.7%	[95.3 - 97.8]	0.58	[0.56 - 0.61]
**Preterm using reported birth timing**	1496	14.8%	[10.6 - 19.9]	96.1%	[94.9 - 97.1]	0.56	[0.53 - 0.58]
By sex							
Male	829	14.4%	[9.0 - 21.3]	96.7%	[95.1 - 97.9]	0.56	[0.53 - 0.59]
Female	667	15.4%	[9.1 - 23.8]	95.4%	[93.4 - 97.0]	0.55	[0.52 - 0.59]
By place of delivery							
Home	692	14.7%	[8.3 - 23.5]	95.2%	[93.1 - 96.7]	0.55	[0.51 - 0.59]
Facility	803	14.9%	[9.6 - 22.6]	97.0%	[95.4 - 98.1]	0.56	[0.53 - 0.59]
By maternal education							
None	1019	12.2%	[7.9 - 17.8]	98.0%	[96.8 - 98.8]	0.55	[0.53 - 0.58]
Any	477	23.6%	[13.2 - 37.0]	92.4%	[89.5 - 94.8]	0.58	[0.52 - 0.64]
By maternal age							
<20 yrs	252	33.3%	[19.1 - 50.2]	94.4%	[90.4 - 97.1]	0.64	[0.56 - 0.72]
20+ yrs	1244	11.3%	[7.3 - 16.4]	96.5%	[95.2 - 97.5]	0.54	[0.52 - 0.56]
By parity							
Primiparous	430	19.7%	[10.9 - 31.3]	92.9%	[89.8 - 95.3]	0.56	[0.51 - 0.61]
2 or more children	1066	13.0%	[8.4 - 18.8]	97.4%	[96.2 - 98.4]	0.55	[0.53 - 0.58]

Table S6: Sensitivity, specificity, AUC for LBW using reported birthweight by binned recall time

Binned Recall Time (months after birth)	Total N	Sensitivity	(95% CI)	Specificity	(95% CI)	AUC	(95% CI)
1 Month	218	45.1%	(31.1 - 59.7)	98.2%	(94.9 - 99.6)	0.72	(0.65 - 0.79)
3 Months	203	61.4%	(47.6 - 74.0)	89.8%	(83.7 - 94.2)	0.76	(0.69 - 0.82)
6 Months	194	34.8%	(21.4 - 50.2)	96.0%	(91.4 - 98.5)	0.65	(0.58 - 0.73)
9 Months	184	45.5%	(32.0 - 59.4)	93.8%	(88.2 - 97.3)	0.70	(0.63 - 0.77)
12 Months	189	53.8%	(39.5 - 67.8)	96.4%	(91.7 - 98.8)	0.75	(0.68 - 0.82)
18 Months	260	39.5%	(29.2 - 50.7)	89.4%	(83.9 - 93.5)	0.65	(0.59 - 0.70)
24 Months	176	34.1%	(20.5 - 49.9)	90.9%	(84.7 - 95.2)	0.63	(0.55 - 0.70)

Table S7: Sensitivity, specificity, AUC, IF for LBW using reported birth size by binned recall time

Binned Recall Time (months after birth)	Total N	Sensitivity	(95% CI)	Specificity	(95% CI)	AUC	(95% CI)
1 Month	222	13.7%	(5.7 - 26.3)	98.3%	(95.0 - 99.6)	0.56	(0.51 - 0.61)
3 Months	207	30.5%	(19.2 - 43.9)	94.0%	(88.8 - 97.2)	0.62	(0.56 - 0.69)
6 Months	201	12.8%	(4.8 - 25.7)	97.4%	(93.5 - 99.3)	0.55	(0.50 - 0.60)
9 Months	191	20.0%	(10.8 - 32.3)	97.7%	(93.5 - 99.5)	0.59	(0.54 - 0.64)
12 Months	193	20.8%	(10.8 - 34.1)	96.4%	(91.9 - 98.8)	0.59	(0.53 - 0.64)
18 Months	280	22.6%	(14.6 - 32.4)	95.8%	(92.0 - 98.2)	0.59	(0.55 - 0.64)
24 Months	192	7.8%	(2.2 - 18.9)	97.2%	(92.9 - 99.2)	0.53	(0.49 - 0.57)

Table S8: Sensitivity, specificity, AUC, IF for preterm using reported birth timing by binned recall time

Binned Recall Time (months after birth)	Total N	Sensitivity	(95% CI)	Specificity	(95% CI)	AUC	(95% CI)
1 Month	221	3.0%	(0.8 - 15.8)	96.8%	(93.3 - 98.8)	0.50	(0.47 - 0.53)
3 Months	206	13.3%	(3.8 - 30.7)	94.9%	(90.6 - 97.6)	0.54	(0.48 - 0.61)
6 Months	202	22.7%	(7.8 - 45.4)	95.6%	(91.5 - 98.1)	0.59	(0.50 - 0.68)
9 Months	193	16.7%	(7.0 - 31.4)	96.7%	(92.5 - 98.9)	0.57	(0.51 - 0.63)
12 Months	192	17.1%	(6.6 - 33.6)	98.7%	(95.5 - 99.8)	0.58	(0.52 - 0.64)
18 Months	283	11.1%	(4.2 - 22.6)	94.0%	(90.2 - 96.7)	0.53	(0.48 - 0.57)
24 Months	199	25.9%	(11.1 - 46.3)	97.1%	(93.3 - 99.0)	0.62	(0.53 - 0.70)

Figure S1: Inflation factors

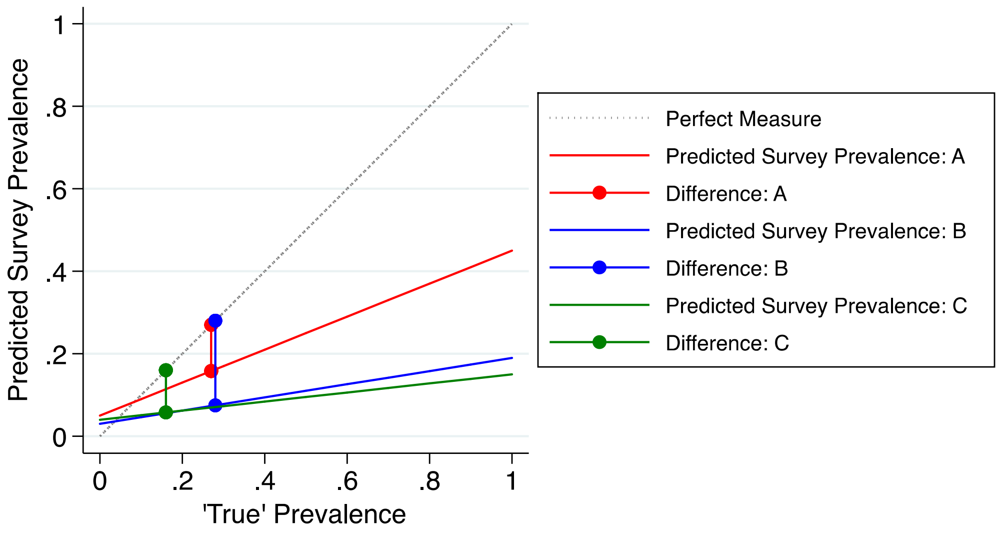

Difference between predicted prevalence based on observed sensitivity and specificity of maternal reports by ‘true’ prevalence of A) LBW using reported birthweight, B) LBW using reported birth size, C) preterm birth using reported length of pregnancy.
